# Calpain Small Subunit 1 Protein in the Prognosis of Cancer Survivors and Its Clinicopathological Correlation

**DOI:** 10.1155/2019/8053706

**Published:** 2019-11-26

**Authors:** Shubin Tang, Qiushi Yin, Fangteng Liu, Yi Zhang

**Affiliations:** ^1^Department of Oncology, The First People's Hospital of Neijiang, Neijiang 641000, Sichuan Province, China; ^2^Department of Hepatobiliary Surgery, The First Affiliated Hospital of Hainan Medical University, Haikou 570100, Hainan Province, China; ^3^Department of Gastrointestinal Surgery, the Second Affiliated Hospital of Nanchang University, Nanchang 330000, Jiangxi Province, China; ^4^Department of General Surgery, The First People's Hospital of Neijiang, Neijiang 641000, Sichuan Province, China

## Abstract

*Background/Aims*. Calpain small subunit 1 (Capn4) is implicated in tumorigenesis and plays a key role in multiple tumors. This study aimed to fully illustrate the prognostic value of Capn4 protein in cancer patients.

**Methods:**

A systematic search was conducted against several online databases. Hazard ratios (HRs) or odds ratio (ORs) were used to investigate the relationship between Capn4 protein expression and prognosis as well as clinical parameters in cancer survivors.

**Results:**

Eleven studies involving 1775 patients were identified. Overall, the results showed that Capn4 protein was associated with poor prognosis of overall survival (OS) (HR=1.74; 95% CI:1.47-2.01;* p*<0.001) and event-free survival (EFS) (HR=1.73; 95% CI:1.39-2.07;* p*<0.001) and event-free survival (EFS) (HR=1.73; 95% CI:1.39-2.07;* p*<0.001) and event-free survival (EFS) (HR=1.73; 95% CI:1.39-2.07;* p*<0.001) and event-free survival (EFS) (HR=1.73; 95% CI:1.39-2.07;* p*<0.001) and event-free survival (EFS) (HR=1.73; 95% CI:1.39-2.07;* p*<0.001) and event-free survival (EFS) (HR=1.73; 95% CI:1.39-2.07;* p*<0.001) and event-free survival (EFS) (HR=1.73; 95% CI:1.39-2.07;* p*<0.001) and event-free survival (EFS) (HR=1.73; 95% CI:1.39-2.07;* p*<0.001) and event-free survival (EFS) (HR=1.73; 95% CI:1.39-2.07;

**Conclusions:**

Expression of Capn4 protein is associated with cancer survival and clinicopathologic characteristics in patients.

## 1. Introduction

Calpain small subunit 1 (Calpain-4; CAPNS1; Capn4), as a member of the calpain family of calcium-dependent cysteine proteases, is a small regulatory subunit (28 kDa) [1]. The calpain family is implicated in regulating a series of cellular processes and plays critical roles in human tumors [2–8]. Recently, an increasing number of studies have reported that Capn4 expression was upregulated in cancer tissues and there were correlations between Capn4 level and clinical outcomes in multiple malignancies, such as colorectal, esophageal, and ovarian cancer [9–11]. However, the potential value of Capn4 protein as a biomarker to predict clinical outcomes is still debatable. For example, Wu et al. [10] reported that elevated expression of Capn4 was linked to poor prognosis as well as lymph node metastasis and deeper tumor invasion in esophageal cancer, which was consistent with the results in nasopharyngeal carcinoma [12], but no significant association was found between Capn4 protein expression and T stage in gastric cancer [13]. And Cheng et al. [9] found that there was a significant relationship between Capn4 protein expression and TNM staging; however, in non-small cell lung cancer, no significant correlation was observed between Capn4 expression and tumor stage [14].

To date, there is no study that systematically assesses the prognostic and pathological value of Capn4 protein expression in survivors and considering the limited sample size of individual study and inconsistent conclusions. Herein, we performed this meta-analysis with published data to explore the relationship of Capn4 protein expression with prognosis and clinicopathological parameters in human cancers and also provide summative insights into the vital roles of Capn4 in tumor prognosis and progression.

## 2. Materials and Methods

### 2.1. Search Strategy and Study Selection

Relevant articles were searched from several databases including PubMed, Embase, Web of Science, and Cochrane library up to May 10, 2018. The following search terms and key-words were used in combination: “calpain small subunit 1” or “CAPNS1” or “CANP” or “CDPS” or “CANPS” or “Capn4” or “Calpain-4”, “cancer” or “tumor” or “neoplasm” or “carcinoma”. Publication language was limited to English. Two authors independently performed the literature retrieval and any disagreements were resolved by discussion together.

In this meta-analysis, all eligible studies had to meet the following selection criteria: (1) studies detected the Capn4 protein expression in human cancer tissue; (2) the relationships between Capn4 protein expression and overall survival (OS) or disease-free survival (DFS) or recurrence-free survival (RFS) or progression-free survival (PFS) were reported; (3) the hazard ratios (HRs) for cancer survival were available; (4) all cases were divided into two groups (H/P and L/N groups) according to the Capn4 protein expression.

The following studies were excluded: (1) those on hematologic malignancies; or animal experiments; or only focused on Capn4 mRNA expression, (2) reviews, editorials, and abstracts, and (3) duplicate articles.

### 2.2. Data Extraction and Quality Assessment

The following data was extracted by two authors, independently: first author's name, publication year, country, number of patients, number of cases in H/P and L/N group, judgement standards for H/P expression, outcome measures, detection methods, analytical methods, and HRs with their 95 % CIs for cancer prognosis. The main clinicopathological features were also extracted from eligible studies: gender, histological grade, depth of tumor invasion, venous invasion, lymph node metastasis (LNM), distant metastasis (DM), and clinical stage. The Newcastle-Ottawa Scale (NOS) was used for quality assessment [15, 16]. A study with NOS scores ≥6 was considered as “high-quality” [16, 17].

### 2.3. Statistical Analysis

In this meta-analysis, STATA statistics software (Version 12.0) and RevMan 5.3 software were used to calculate the pooled hazard ratios (HRs) and odds ratios (ORs) with their 95% CIs, respectively. DFS, PFS, and RFS were merged as event-free survival (EFS) [18, 19]. Statistical heterogeneity was evaluated by the Q-statistic test and I^2^ statistic test, and two different models were selected according to the heterogeneity level; if there was significant heterogeneity (P_Q_ < 0.1 or/and I^2^>50%), then the random-effects model was used; otherwise, the fixed-effects model was applied for nonsignificant heterogeneity. The visible plot and Begg's test were used to assess the potential publication bias, and the robustness of pooled data was assessed by sensitivity analysis omitting each one study sequentially. A* p*< 0.05 was considered to be statistically significant.

## 3. Results

### 3.1. Eligible Studies and Basic Characteristics

**Figure 1:**
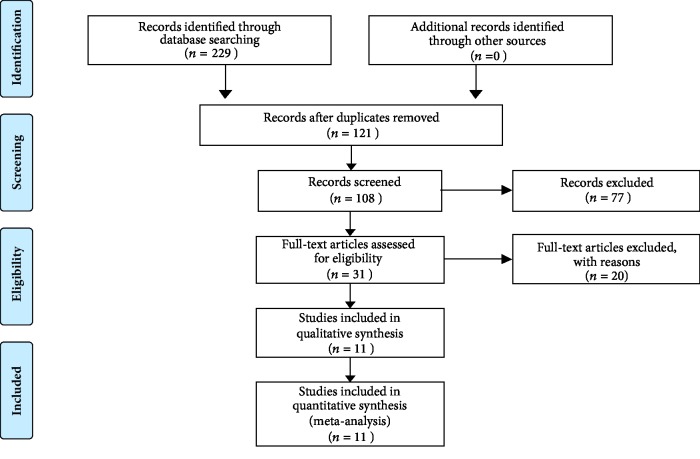
Flow diagram of included studies for the meta-analysis.

According to the selection criteria, eleven studies [9–14, 20–24] were finally included into this meta-analysis, all included studies were retrospective studies from China, and a total of 1775 patients were recruited in this work. The sample size varied from 91 to 323. All studies detected the Capn4 protein expression in tissue samples using immunohistochemistry (IHC) method, and several evaluation criteria were applied among these studies, including by the extent of stained cells or the area of positive tumor cells or by multiplying the ratio of positive cells score and the intensity score. The detailed evaluation criteria for these studies were listed in Table 1. All included studies investigated the relationships between Capn4 protein expression and cancer survival, including 11 studies for OS, 1 covered DFS, 2 reported RFS, and 3 reported PFS. For calculating the pooled HRs of Capn4 protein, DFS, PFS, and RFS were integrated into the meta-analysis of EFS, and the follow-up time in all cohort studies was equal or more than 5 years. As for cancer types, 8 different kinds of cancers were investigated, including colorectal cancer (CRC), esophageal squamous cell carcinoma (ESCC), ovarian cancer (OC), intrahepatic cholangiocarcinoma (ICC), nasopharyngeal carcinoma (NPC), non-small cell lung cancer (NSCLC), glioblastoma (GBM), and hepatocellular carcinoma (HCC). The procedure of the literature search is shown in Figure 1. Main characteristics of included studies are presented in Table 1.

### 3.2. Capn4 Protein and OS

**Figure 2:**
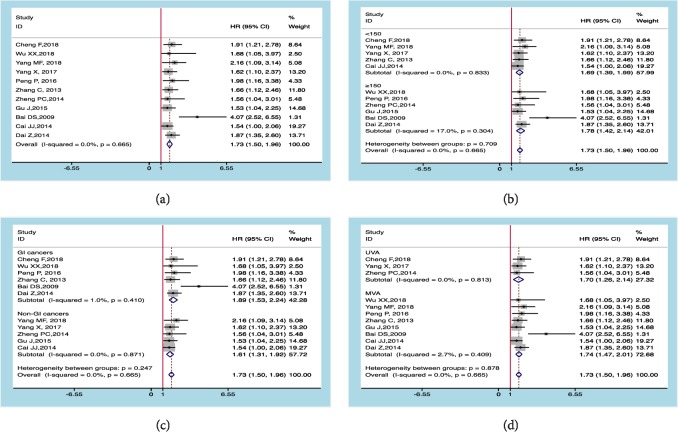
Meta-analysis of Capn4 protein and overall survival (OS). (a) Overall; (b) by sample size; (c) by cancer types; (d) by analysis methods.

As presented in Figure 2(a), the fixed-effects model was applied for no significant heterogeneity existed (I^2^ = 0.0%; P_Q_ = 0.665); the pooled HR value of OS merged in the eleven eligible articles was 1.73 with the corresponding 95% CI 1.50-1.96 and* p* < 0.05, suggesting that high level of Capn4 protein, as a prognostic factor of cancer survivors, indicated a shorter OS. Furthermore, the prognostic values of Capn4 protein were also confirmed in several subgroups (Figures 2(b)–2(d), Table 2). Interestingly, the Capn4 protein could be an independent predictor for OS in cancers (HR=1.74; 95% CI:1.47-2.01).

### 3.3. Capn4 Protein and Event-Free Survival (EFS)

**Figure 3:**
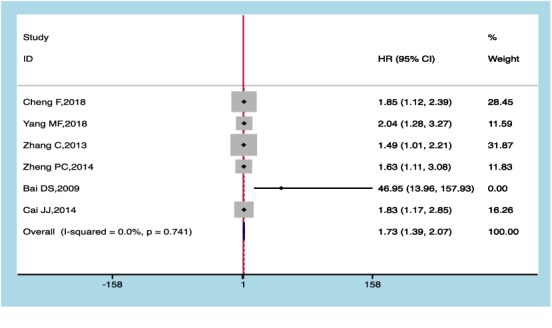
Meta-analysis of Capn4 protein and event-free survival (EFS).

As shown in Figure 3, no heterogeneity existed for values of I^2^ = 0.0% and P_Q_ = 0.744, and the combined HR value of the EFS rate was 1.73 with the corresponding 95% CI:1.39-2.07 and* p* < 0.05 after being merged in the six included studies, indicating that high level of Capn4 protein may lead to an inferior EFS in cancer patients.

### 3.4. Capn4 Protein and Clinicopathological Characteristics

**Figure 4:**
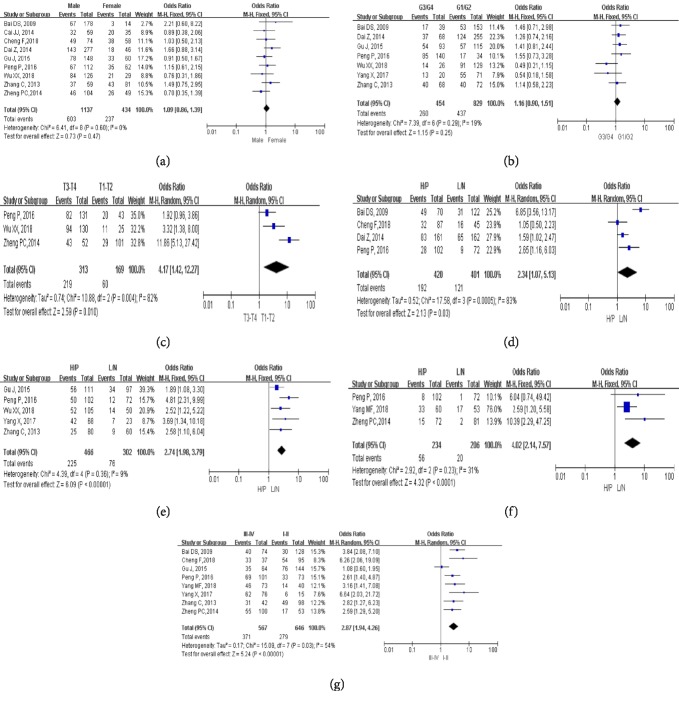
Meta-analysis of Capn4 protein and clinicopathological features: (a) gender; (b) histological grade; (c) depth of tumor invasion; (d) venous invasion; (e) lymph node metastasis; (f) distant metastasis; (g) clinical stage.

The pooled ORs for Capn4 protein and clinicopathological relevance are presented in Figures 4(a)–4(g) and Table 3. No significant associations were observed between Capn4 protein and gender (OR=1.09; 95% CI: 0.86-1.39; Figure 4(a)) and histological grade (OR=1.16; 95% CI: 0.90-2.23; Figure 4(b)). However, increased expression level of Capn4 protein was significantly related to deeper tumor invasion (OR= 4.17; 95% CI: 1.42-12.27; Figure 4(c)), venous invasion (OR=2.34; 95% CI: 1.07-5.13; Figure 4(d)), and high rate of metastases (LNM: OR= 2.74; 95% CI: 1.98-3.79; Figure 4(e); DM: OR=4.02; 95% CI: 2.14-7.57; Figure 4(f)) and advanced clinical stage (OR=2.87; 95% CI: 1.94-4.26; Figure 4(g)).

### 3.5. Publication Bias

**Figure 5:**
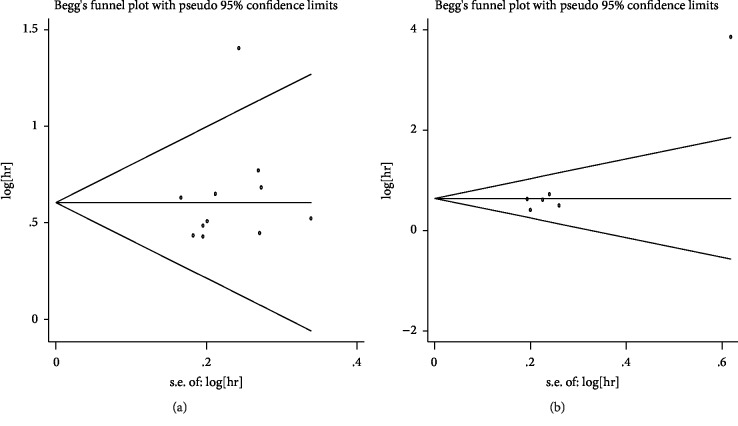
Publication bias assessment for OS (a) and EFS (b).

Begg's plots were shown in Figures 5(a)-5(b); the results of Begg's tests indicated no significant publication bias in this meta-analysis (OS: Pr > |z| = 0.119 (continuity corrected); EFS: Pr > |z| = 0.452 (continuity corrected)).

### 3.6. Sensitivity Analysis

**Figure 6:**
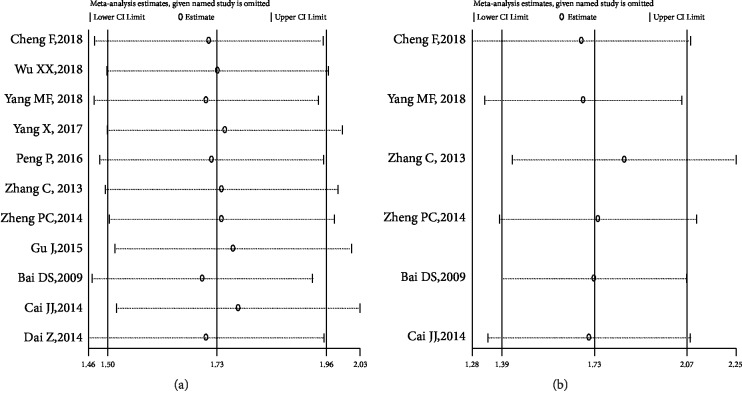
Sensitivity analysis for OS (a) and EFS (b).

Sensitivity analysis was performed (Figures 6(a)-6(b)) and confirmed the robustness of the pooled results.

## 4. Discussion

Despite great progress in supervision and treatment strategies in recent decades, the long-term prognosis of malignant tumors remains disappointing [25–27]. On the other hand, to classify the patients with a high possibility of tumor recurrence and predict the probable clinical outcome could significantly help to timely initiate intervention and select optimized treatment plans [26, 27]. Thus, it is essential to identify effective tumor markers associated with progression and survival to improve the prognosis of cancer survivors.

Our data indicated that the expression of Capn4 protein was closely related with the survival of cancer patients. In this meta-analysis, eleven studies with 1775 patients were enrolled to assess the prognostic value of OS in cancer survivors, and the combined data showed that high expression level of Capn4 protein was significantly associated with the poor long-term OS. Particularly, the Capn4 protein could be a valuable prognostic factor of OS in gastrointestinal (GI) cancers and also might serve as an independent factor for OS in cancers. Meanwhile, the data from six studies with a total of 824 cases were also combined to investigate the relationship between Capn4 protein and EFS, and Capn4 protein was suggested to be a useful prognostic indicator for EFS.

There are some clues that might help to explain why Capn4 could predict the survival of cancer patients as well as the tumor progression. Many scholars presented that Capn4 was frequently highly expressed in cancer tissues and tumor cell lines [23, 24, 28]. Accumulating evidence supports that Capn4 acts as an oncogene in various human cancers, such as ovarian carcinoma, cholangiocarcinoma, and liver cancer, and the elevated expression of Capn4 also indicates malignant biological behaviors in tumors [20–23]. Capn4 plays an important role in the occurrence and progression of cancers. Capn4 knockdown could significantly inhibit tumor growth, invasion, and metastasis in vitro and in vivo. Conversely, Capn4 upregulation could enhance cell growth, metastasis, and tumor transformation [23–25, 29]. What is more important, it is reported that Capn4 is involved in tumor progression through many pathways, such as epithelial-mesenchymal transition (EMT), the FAK–Src signaling pathways, the Wnt/*β*-catenin (*β*-catenin), and the nuclear factor *κ*B (NF-*κ*B) signaling pathways [24, 30–32]. Additionally, Capn4 also correlates with drug response and tumor resistance and might serve as a potential therapeutic target for several kinds of tumors [13, 14, 33, 34].

To further assess the clinical relevance of Capn4 protein, we also linked it to clinicopathological characteristics of cancers. The pooled data showed that the overexpression of Capn4 protein was significantly associated with deeper tumor invasion, venous invasion, positive nodal status, and distant tumor metastasis. The patients with high expression Capn4 protein were more likely to have an advanced tumor stage. All the results showed that Capn4 predicted worse pathological features, which was consistent with the OS/EFS analysis.

Overall, Capn4 exhibited the powerful clinical usefulness of Capn4 as a promising biomarker in cancer survivors, and the results of this cumulative meta-analysis provided a clue toward understanding the potential clinical utility of Capn4 as an unfavorable predictor as well as a new therapeutic target in cancers. The Capn4 protein expression in patient samples could be determined by the IHC, a widely used and robust primary technique, which could make our findings translating into clinical applications more easily. Certainly, the measurement of the expression of Capn4 protein should be verified with an established standard manipulation procedure, including consistent sample processing and incubation time, and the IHC evaluation criteria also need to be unified. In addition, more researches that explore the mechanism of the role of Capn4 in cancers are required to draw major clinical conclusions.

In the current study, several limitations should be acknowledged. First, the total sample size and included studies were relatively small, and only eleven studies were included with 1775 patients. Second, studies from other countries should be included in the future, the patients enrolled in this work were all from China, and this might limit our results applicable to other ethnics. Third, all studies included were retrospectively designed. Fourth, the clinical potential of Capn4 protein in certain specific cancers needed further investigation. Fifth, the heterogeneity was also observed in the quantified synthesis, especially for the relationship between Capn4 expression and invasion depth, venous invasion, and clinical stage. Finally, the standards for Capn4 protein overexpression in tissues varied in these studies.

In conclusion, this meta-analysis provides meaningful statistical evidence supporting the important prognostic significance of Capn4 in cancer survivors. It demonstrates the associations between high Capn4 expression and poor clinical outcomes for cancer patients. Large-scale studies with high-quality from multicenter are needed to verify the clinical application of Capn4 protein as a prognostic marker in cancers.

## Figures and Tables

**Table 1:** Characteristics of eligible studies in this meta-analysis.

Author, Year	Cancer type	Total number	Calpain-4 protein expression		Judgment standards for high Capn4 expression	Outcome measures	Follow-ups	Analysis	HR (95%CI) for OS	HR (95%CI) for DFS/PFS/RFS	NOS
			H/P	L/N							
Cheng F, 2018	CRC	132	87	45	Each specimen was scored for the extent of stained cells (0-1% = 0, 1~24% =1, 25~49% =2, 50~74% =3, 75~100% =4). A value ≥2 as a high score.	OS, DFS	≥5 years	UVA	1.91 (1.21-2.78)	1.85 (1.12-2.39)	6

Wu XX, 2018	ESCC	155	105	50	The final score was obtained by multiplying the ratio of positive cells score and the intensity score, and a final score of 8-12 was classified as high expression.	OS	≥5 years	MVA	1.68 (1.05-3.97)	NR	7

Yang MF, 2018	OC	113	60	53	The final scores of Capn4 expression, ranging from 0 to 9, were calculated by multiplying the percentage score by the intensity score. A final score of ≥4 was classified as high expression.	OS, PFS	≥5 years	MVA	2.16 (1.09-3.14)	2.04 (1.28-3.27)	7

Yang X, 2017	OC	91	68	23	The staining index was evaluated by multiplying the score of staining intensity and the percentage of positive tumor cells, samples with an SI ≥8 were defined as showing high expression.	OS	≥5 years	UVA	1.62 (1.10-2.37)	NR	6

Peng P, 2016	GC	174	102	72	Composite expression scores = 4 (intensity score−1) + frequency score. CES of calpain-4 greater than or equal to 6 were considered to be high expression.	OS	≥5 years	MVA	1.98 (1.16-3.38)	NR	8

Zhang C, 2013	ICC	140	80	60	Capn4 high: the mean area of positive staining>50% of the tumor section.	OS, RFS	≥5 years	MVA	1.66 (1.12-2.46)	1.49 (1.01-2.21)	7

Zheng PC, 2014	NPC	153	72	61	The scores of 0, 1-2, 3-4, and 5-6 were considered to be negative, low, medium, and strong, respectively. The scores of 5–6 were considered to be high.	OS, PFS	≥5 years	UVA	1.56 (1.04-3.01)	1.63 (1.11-3.08)	7

Gu J, 2015	NSCLC	208	111	97	The intensity of Capn4 was classified into four grades (0 for negative; 1 for weak; 2 for moderate; and 3 for strong). Scores of 2 or 3 were considered Capn4 high.	OS	≥5 years	MVA	1.53 (1.04-2.25)	NR	8

Bai DS, 2009	HCC	192	70	122	The final score of each sample was assessed by summarizing the result of intensity and extent of staining. The case was considered positive if the final score was 4 to 5 (+) or 6 to 7 (++).	OS, RFS	≥5 years	MVA	4.07 (2.52-6.55)	46.95 (13.96-157.93)	8

Cai JJ, 2014	GBM	94	52	42	A positive reaction for Capn4 was classified into four grades (0 for negative; 1 for weak; 2 for moderate; and 3 for strong). The moderate or strong intensity was classified as high Capn4 expression.	OS, PFS	≥5 years	MVA	1.54 (1.00-2.06)	1.83 (1.17-2.85)	6

Dai Z, 2014	HCC	323	161	162	NR	OS	≥5 years	MVA	1.87 (1.35-2.60)	NR	8

CRC: colorectal cancer; ESCC: esophageal squamous cell carcinoma; OC: ovarian cancer; ICC: intrahepatic cholangiocarcinoma; NPC: nasopharyngeal carcinoma; NSCLC: non-small cell lung cancer; GBM: glioblastoma; HCC: hepatocellular carcinoma; IHC: immunohistochemistry; H/P: high/positive expression; L/N: low/negative expression; OS: overall survival; DFS: disease-free survival; RFS: recurrence-free survival; PFS: progression-free survival; UVA: univariate analysis; MVA: multivariate analysis; SI: staining index; CES: composite expression scores; NR: not reported.

**Table 2:** Subgroup analysis of the association between Capn4 protein expression and OS.

Subgroup factor	Divided standard	No. of studies	Pooled HR	*p*-value	Heterogeneity	
			(95% CI)		I^2^ (%)	P_Q_
Sample size	≥ 150	6	1.78(1.42-2.14)	<0.001	17.0	0.304
	< 150	5	1.69(1.39-1.99)	<0.001	0.0	0.833
Cancer type	GI cancers	6	1.89(1.53-2.24)	<0.001	1.0	0.410
	Non-GI cancers	5	1.61(1.31-1.92)	<0.001	0.0	0.871
Analysis method	MVA	8	1.74(1.47-2.01)	<0.001	2.7	0.409
	UVA	3	1.70(1.26-2.14)	<0.001	0.0	0.813

GI: Gastrointestinal; UVA: univariate analysis; MVA: multivariate analysis; HR: hazard ratios.

**Table 3:** Results of the meta-analysis of Capn4 protein and clinicopathological features.

Parameters	Studies	Number of cases	OR (95% CI)	*p*-value	Heterogeneity		
	(n)				I^2^ (%)	P_Q_	Model
Gender (Male vs. Female)	9	1571	1.09(0.86-1.39)	0.47	0.0	0.60	Fixed
Histological grade (G3/G4 vs. G1/G2)	7	1283	1.16(0.90-2.23)	0.25	19	0.29	Fixed
Tumor depth (T3-4 vs. T1-2)	3	482	4.17(1.42-12.27)	0.01	82	0.004	Random
Venous invasion (+ vs. -)	4	821	2.34(1.07-5.13)	0.03	83	0.0005	Random
LNM (+ vs. -)	5	768	2.74(1.98-3.79)	<0.001	9	0.36	Fixed
DM (+ vs. -)	3	440	4.02(2.14-7.57)	<0.001	31	0.23	Fixed
Clinical stage (III-IV vs. I-II)	8	1213	2.87(1.94-4.26)	<0.001	54	0.03	Random

LNM: lymph node metastasis; DM: distant metastasis; OR: odds ratio.

## Data Availability

The data used to support the findings of this study are included within the article.
